# Endoscopic balloon tamponade for massive bleeding from aorto-esophageal fistula: a simple technique for rapid hemostasis

**DOI:** 10.1055/a-2727-0284

**Published:** 2025-11-10

**Authors:** Yuichiro Mikuriya, Kazuo Shiotsuki, Takanori Hama, Nobuhisa Minakata, Kohei Takizawa, Shin Maeda

**Affiliations:** 1 91321 Department of Gastroenterology, Kanagawa Cancer Center, Yokohama, Japan; 2 26438 Department of Gastroenterology, Yokohama City University Graduate School of Medicine, Yokohama, Japan

An aorto-esophageal fistula after chemoradiotherapy (CRT) for esophageal cancer is fatal 1 2 . Early diagnosis and prompt intervention are required for survival 3 . Endoscopic approaches have been sporadically reported; however, a standardized strategy has not been established 4 5 . Herein, we present a case in which lifesaving hemostasis was achieved by compressive tamponade using a through-the-scope (TTS) dilation balloon and provide practical tips for its use ( Video 1 ).

Utility of endoscopic balloon tamponade for refractory acute esophageal bleeding due to aorto-esophageal fistula.Video 1

A 62-year-old man was diagnosed with advanced esophageal squamous cell carcinoma (cT4N2M0) 6 months prior and received CRT.

The patient was admitted to our hospital with hematemesis, and urgent esophagogastroduodenoscopy showed a residual tumor and luminal narrowing with an adherent blood clot. After removal of the large clot, a massive spurting life-threatening hemorrhage from the residual lesion occurred. Thermal and mechanical hemostatic methods (e.g., coagulation forceps and clips) were considered impractical due to poor visualization and spurting bleeding. Therefore, we first compressed the bleeding point with an endoscope to clear the field, and then introduced a CRE Fixed Wire dilation balloon (12–15 mm; Boston Scientific, Marlborough, MA, USA). The balloon was inflated within the esophageal lumen, producing sustained tamponade and immediate hemostasis ( Fig. 1 ). Rebleeding occurred whenever the balloon was deflated; therefore, balloon tamponade was maintained while contrast-enhanced computed tomography was performed, confirming an aorto-esophageal fistula at the site of the tumor ( Fig. 2 ). As definitive therapy was unavailable locally, the balloon remained inflated during transfer to a tertiary center, where thoracic endovascular aortic repair was successfully performed, and the patient survived. TTS balloon tamponade requires no specialized expertise, utilizes widely available equipment, and can achieve prompt and effective primary hemostasis, thereby serving as a bridge to definitive vascular interventions. This simple and readily implementable technique can help clinicians in life-threatening situations.

**Fig. 1:**
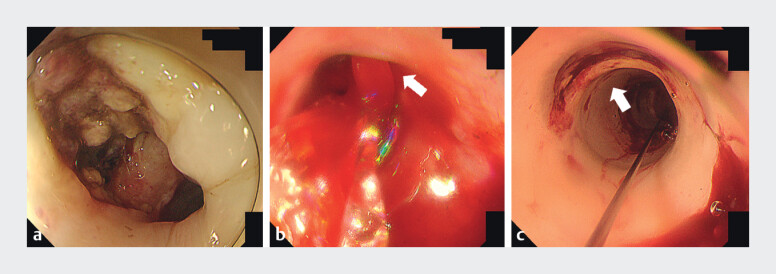
Endoscopic balloon tamponade technique. **a** Residual tumor with an adherent blood clot, as seen on esophagogastroduodenoscopy. **b** Massive hemorrhage after clot removal. **c** Immediate hemostasis achieved with balloon tamponade.

**Fig. 2:**
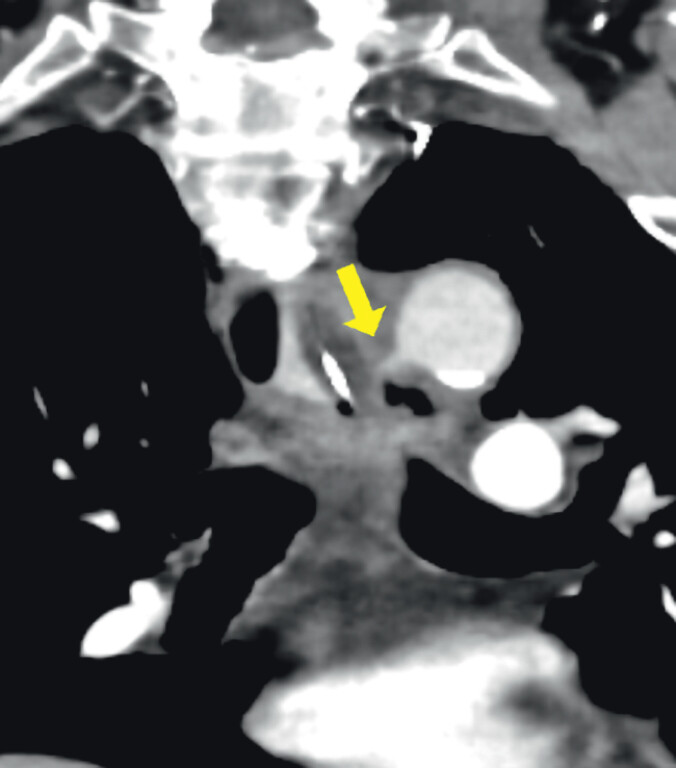
Contrast-enhanced computed tomography showing an aorto-esophageal fistula at the site of the tumor.

Endoscopy_UCTN_Code_TTT_1AO_2AD

## Notes

Endoscopy E-Videos https://eref.thieme.de/e-videos
*E-Videos* is an open access online section of the journal *Endoscopy* , reporting on interesting cases and new techniques in gastroenterological endoscopy. All papers include a high-quality video and are published with a Creative Commons CC-BY license. Endoscopy E-Videos qualify for HINARI discounts and waivers and eligibility is automatically checked during the submission process. We grant 100% waivers to articles whose corresponding authors are based in Group A countries and 50% waivers to those who are based in Group B countries as classified by Research4Life (see: https://www.research4life.org/access/eligibility/ ). This section has its own submission website at https://mc.manuscriptcentral.com/e-videos .
